# Synthesis and Characterization of Modified BiOCl and Their Application in Adsorption of Low-Concentration Dyes from Aqueous Solution

**DOI:** 10.1186/s11671-018-2480-y

**Published:** 2018-03-01

**Authors:** Qihang Zhao, Yongxing Xing, Zhiliang Liu, Jing Ouyang, Chunfang Du

**Affiliations:** 1 College of Chemistry and Chemical Engineering, Inner Mongolia University, Hohhot, 010021 Inner Mongolia People’s Republic of China; 2 Hunan Key Laboratory of Mineral Materials and Application, Central South University, Changsha, 410083 People’s Republic of China

**Keywords:** Bismuth oxychloride, Modification, Adsorption, Low concentration

## Abstract

The synthesis and characterization of BiOCl and Fe^3+^-grafted BiOCl (Fe/BiOCl) is reported that are developed as efficient adsorbents for the removal of cationic dyes rhodamine B (RhB) and methylene blue (MB) as well as anionic dyes methyl orange (MO) and acid orange (AO) from aqueous solutions with low concentration of 0.01~0.04 mmol/L. Characterizations by various techniques indicate that Fe^3+^ grafting induced more open porous structure and higher specific surface area. Both BiOCl and Fe/BiOCl with negatively charged surfaces showed excellent adsorption efficiency toward cationic dyes, which could sharply reach 99.6 and nearly 100% within 3 min on BiOCl and 97.0 and 98.0% within 10 min on Fe/BiOCl for removing RhB and MB, respectively. However, Fe/BiOCl showed higher adsorption capacity than BiOCl toward ionic dyes. The influence of initial dye concentration, temperature, and pH value on the adsorption capacity is comprehensively studied. The adsorption process of RhB conforms to Langmuir adsorption isotherm and pseudo-second-order kinetic feature. The excellent adsorption capacities of as-prepared adsorbents toward cationic dyes are rationalized on the basis of electrostatic attraction as well as open porous structure and high specific surface area. In comparison with Fe/BiOCl, BiOCl displays higher selective efficiency toward cationic dyes in mixed dye solutions.

**Electronic supplementary material:**

The online version of this article (10.1186/s11671-018-2480-y) contains supplementary material, which is available to authorized users.

## Background

A serious environmental hazard, caused by water pollution with toxic dyes, organic contaminants, and metal ions, has come to the public notice [1–4]. The discharged wastewater is mostly toxic, non-biodegradable, and dangerous to human health and marine organisms. Therefore, the pollutants must be removed from the wastewater to solve the biological, ecological, and environmental problems. Various techniques, including ion exchange [5], adsorption [6, 7], chemical precipitation [8], advanced oxidation [9–11], biodegradation [12, 13], and photocatalysis [14–16] have been tried to the pollutant removal in the wastewater. The adsorption method is easily handled, highly efficient, economically feasible, and environmentally friendly [17–19], which is therefore considered as a competitive route for efficiently removing the pollutants from wastewater.

Bismuth oxychloride (BiOCl), as a photocatalyst, has recently drawn a lot of attention [20–25]. However, its excellent adsorption capacity toward dyes or organic pollutants in wastewater alerts little attention [26–28]. As is known to all, the adsorption capacity is heavily influenced by the morphology, particle size, and composition of an adsorbent [29]. The representative morphology of BiOCl is a three-dimensional (3D) hierarchical flower-like microstructure. This specific porous structure and large surface area of the 3D hierarchical flower-like morphology is extensively beneficial for the adsorption process [28, 30, 31]. Surface modification is a universal technique to improve the adsorption capacity of an adsorbent. Yu et al. [32] improved the adsorption capacities of BiOCl toward dyes congo red (CR) and reactive red 3 (X3B) through attaching CTAB on the outside of BiOCl, which could reach the maximum adsorption capabilities of 901 and 699 mg/g for CR and X3B, respectively. Sohn [29] improved the adsorption capacities of BiOI for removing dyes of methyl orange (MO), rhodamine B (RhB), and methylene blue (MB) via a Ti-loading route. The adsorption capability of BiOCl could be also enhanced by introducing iodine, which reached the maximum adsorption value toward hydroxyphenylacetic acid (p-HPA) when the I/Cl molar ratio was 0.5 and decreased with further increasing of the I/Cl molar ratio [33].

In this work, we successfully synthesized 3D hierarchical BiOCl microstructure via an Fe^3+^-modified method. Cationic dyes (RhB and MB) and anionic dyes (MO and acid organic, AO) with low concentration of 0.01~0.04 mmol/L are respectively chosen to check the adsorption efficiencies of as-synthesized BiOCl and Fe^3+^-grafted BiOCl (Fe/BiOCl) for the first time, although their photocatalytic performances have been reported [34]. Their mixed dye adsorption efficiencies were also studied. Furthermore, the influence of various reaction parameters, including pH value, reaction temperature, and initial concentration, on the adsorption capacities of BiOCl and Fe/BiOCl were discussed. To thoroughly understand the adsorption process, adsorption isotherm and kinetic feature were investigated and a relationship between adsorbent structure and dye adsorption capacity was proposed. This work not only provides a new idea for constructing an adsorbent with enhanced adsorption capability, but also is beneficial for better understanding the relationship between adsorbent structure and dye adsorption capacity.

## Methods

### Synthesis of BiOCl and Fe/BiOCl

Analytical grade chemicals of Bi(NO_3_)_3_·5H_2_O, Fe(NO_3_)_3_·9H_2_O, KCl, and glycerol were purchased from Shanghai Chemical Industrial Co., all of which were used as the starting materials without further purification.

In a typical procedure of BiOCl, 0.776 g Bi(NO_3_)_3_·5H_2_O was dissolved in 76 mL of glycerol with magnetic stirring (solution A), and 0.12 g KCl was dissolved in 4 mL of deionized water (solution B). Subsequently, the obtained KCl solution was mixed with solution A and transferred into a Teflon-lined stainless-steel autoclave. The autoclave was heated at 110 °C and kept at this temperature for 8 h. The resulting precipitate was collected by centrifugation, washed with ethanol, and deionized water for several times and dried at 80 °C. Finally, the powder was calcined at 400 °C to obtain the pure BiOCl powder. The preparation process of Fe/BiOCl was the same as that of BiOCl, except for the addition of various amount of Fe(NO_3_)_3_·9H_2_O in solution A. The final products were denoted as Fe/BiOCl (*x*), where *x* represented the molar ratio of Fe/Bi.

### Characterization

X-ray powder diffraction (XRD) patterns were recorded on an X-ray diffractometer (Empypeanp Panalytical) with Cu Kα radiation (*λ* = 0.154 nm). The detailed morphologies and structures were conducted by transmission electron microscopy (TEM) and high-resolution TEM (HRTEM) on a JEM-2010 microscope operated at 200 kV. Scanning electron microscopy (SEM) images were recorded on the Hitachi S-4800 apparatus with an accelerating voltage of 15 kV. The chemical compositions and surface states of the samples were analyzed by X-ray photoelectron spectroscopy (XPS), which were carried out on a Thermo Escalab 250Xi photoelectron spectrometer with a monochromatic Al Kα (*hv* = 1486.6 eV). The N_2_ adsorption-desorption isotherms were measured at 77 K operated at a Micrometrics ASAP 2020. Prior to the measurement, the as-synthesized samples were degassed in a vacuum at 180 °C for 8 h. The specific surface areas were calculated by the Brunauer-Emmett-Teller (BET) method; the pore size distributions of samples were derived from the desorption branches of isotherms by using Barrett-Joyner-Halenda (BJH) model. The zeta potential of samples was measured using DelsaTM Nano Zeta Potential to check the surface charge at different pH values.

### Adsorption Capacity Test

The adsorption experiments were carried out in the dark at room temperature. Cationic dyes of MB and RhB and anionic dyes of MO and AO were selected as the typical organic dyes to check the adsorption capacities of BiOCl and Fe/BiOCl. In a typical adsorption experiment, 50 mg as-prepared sample was respectively added into 50 mL of various dye solutions with different concentrations ranging from 0.01~0.04 mmol/L under magnetic stirring. At each given time interval, 3 mL of suspension was taken out and centrifuged to remove the solid powder. The concentration of remaining dyes was determined using a UV-vis spectrophotometer (Hitachi U-3900).

The residual dye percentage can be calculated using Eq. (1):

1 $$ \mathrm{residual}\ \mathrm{percentage}\ \left(\%\right)=\frac{C_{\mathrm{t}}}{C_0}\times 100\% $$

The amount of dye molecules adsorbed at time *t* was calculated using Eq. (2):

2 $$ {q}_t=\frac{\left({C}_0-{C}_t\right)V}{m} $$

where *C*_*0*_ and *C*_*t*_ (mg/L) are the concentration of dye molecules at initial and any time *t*, respectively; *q*_*t*_ is the amount of dye molecules adsorbed on per unit of adsorbent at time *t* (mg/g); *V* is the volume of dye solution (*L*); and *m* is the weight of adsorbent (mg).

Effects of the experimental parameters, including adsorption time, initial dye concentration, temperature, and pH value were studied to optimize the adsorption process.

The recyclability of BiOCl and Fe/BiOCl adsorbents was also conducted. For the dye desorption, 50 mg of BiOCl and Fe/BiOCl was added into the 50 mL of NaOH ethanol solution (0.01 M) and then was stirred for 60 min, respectively. Subsequently, the adsorbent was collected, washed thoroughly with water, and dried. The obtained product was then used for adsorption in the next adsorption cycle.

## Results and Discussion

### Material Characterization

**Fig. 1:**
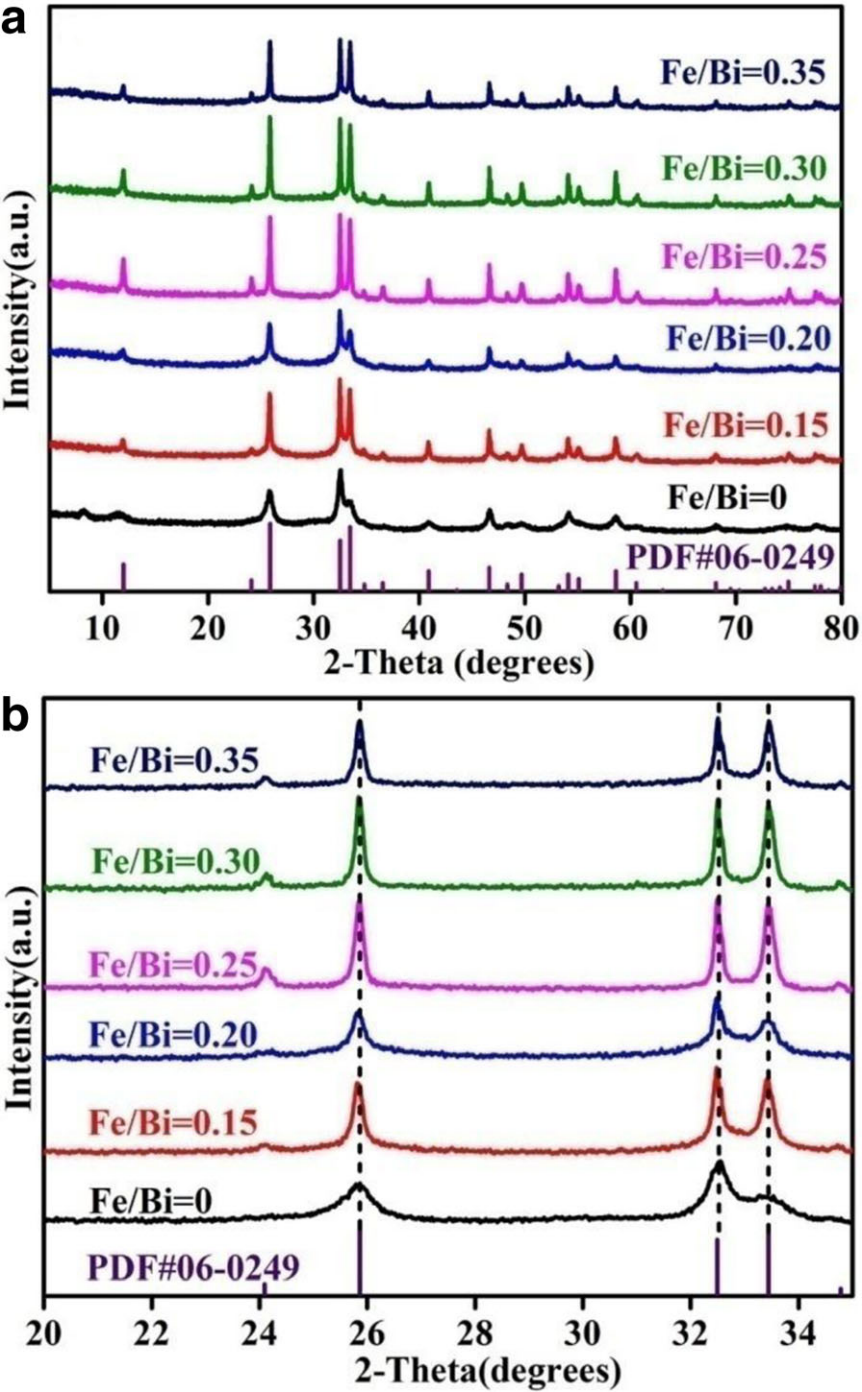
Wide XRD patterns (**a**) and local XRD patterns (**b**) of Fe/BiOCl (*x*)

Figure 1a shows the XRD patterns of BiOCl with different Fe/Bi molar ratios. All peaks of pure BiOCl (Fe/Bi = 0) are in good agreement with the tetragonal BiOCl (JCPDS 06-0249), and no other XRD peaks are observed. With an increase of Fe/Bi molar ratio, the XRD peaks become stronger and sharper. It is noticed that no new peaks are observed in the range of 2*θ* = 20~35° (Fig. 1b). As known, Fe^3+^ could easily hydrolyze to form (hydr)oxides which gradually convert to crystalline iron oxides [35, 36]. However, no diffraction peaks corresponding to iron oxides are observed in the XRD patterns of Fe/BiOCl (*x*), i.e., iron oxides did not form in our samples although the samples had a thermal treatment at 400 °C for 3 h. Moreover, the characteristic peaks of Fe/BiOCl (*x*) have no shifts compared with those of pure BiOCl, which indicates that Fe^3+^ ions are not incorporated into the crystal lattice of BiOCl [37, 38]. Thus, it could be concluded that the irons are mostly present as highly dispersed Fe^3+^ form rather than iron oxides or doping ions on the surface or in the crystal lattice of BiOCl, which result is in accordance with that of Cu/BiOCl [39] and Fe(III)-BiOCl [34].

**Fig. 2:**
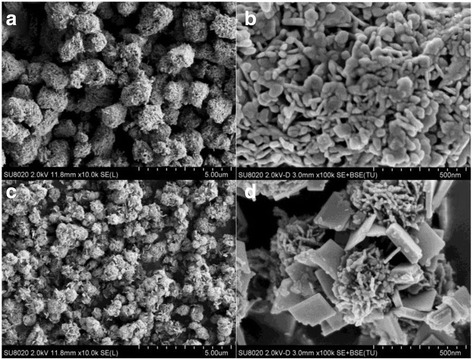
SEM images of BiOCl (**a**, **b**) and Fe/BiOCl (0.25) (**c**, **d**)

The representative SEM images of Fe/BiOCl (*x*) are shown in Fig. 2. It can be clearly seen from Fig. 2a that BiOCl displays a 3D microsphere-like structure with an average diameter of about 1~2 μm. The high-magnification SEM image (Fig. 2b) reveals that BiOCl microspheres are tightly assembled by numerous irregular nanoplates with a width of about 70 nm and a thickness of about 20 nm. After Fe^3+^ grafting, the morphology of Fe/BiOCl remains the sphere-like structure but displays the decreased diameter of about 0.5~1 μm (Fig. 2c). From the high-magnification SEM image of Fe/BiOCl (Fig. 2d), it could be found that Fe/BiOCl microspheres are composed of plenty of nanosheets with a thinner thickness of about 15 nm. Furthermore, some new square-like nanosheets are also observed. As reported, the introduction of Fe^3+^ ions could induce a morphology transformation in bismuth oxyhalides [11, 37, 40]. The existence of Fe^3+^ in our samples possibly acts a role for inducing a hierarchical microstructure with thinner nanosheets.

**Fig. 3:**
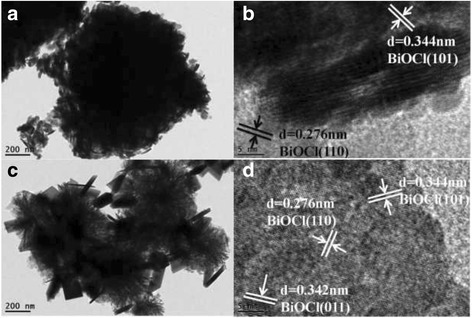
TEM and HRTEM images of BiOCl (**a**, **b**) and Fe/BiOCl (0.25) (**c**, **d**)

The geometrical structure and morphology of samples were further investigated by TEM and HRTEM techniques. The typical TEM image (Fig. 3a) of an individual structure further reveals the sphere-like morphology of BiOCl with a diameter of about 1 μm, which is constructed by nanoplates displaying approximately round edges with a thickness of 20 nm. Figure 3b shows the HRTEM image of the thin nanoplates, where the clear lattice fringes indicate the good crystallinity and single-crystal nature of these plate-like subunits. The lattice fringes with *d* spacing of 0.276 nm belong to the (110) crystalline plane of BiOCl. Other lattice fringes with *d* spacing of 0.344 nm correspond to the (101) planes of BiOCl. In comparison with pure BiOCl, the Fe/BiOCl sample is composed of hierarchical micro-flowers loosely assembled by substantial nanosheets with a small quantity of square-like structures, which result is consistent with the SEM result (Fig. 2c). The lattice fringes in Fig. 3d with *d* spacing of 0.276, 0.344, and 0.342 nm belong to the (110), (101), and (011) crystal plane of BiOCl, respectively. Based on the SEM and TEM results, it could be deduced that Fe^3+^ ions can induce the regular square-like nanosheet growth and drive the nanosheets to form hierarchical micro-flowers with open porous structure, which transformation could be possibly related to the oriented attachment and Ostwald ripening [37].

**Fig. 4:**
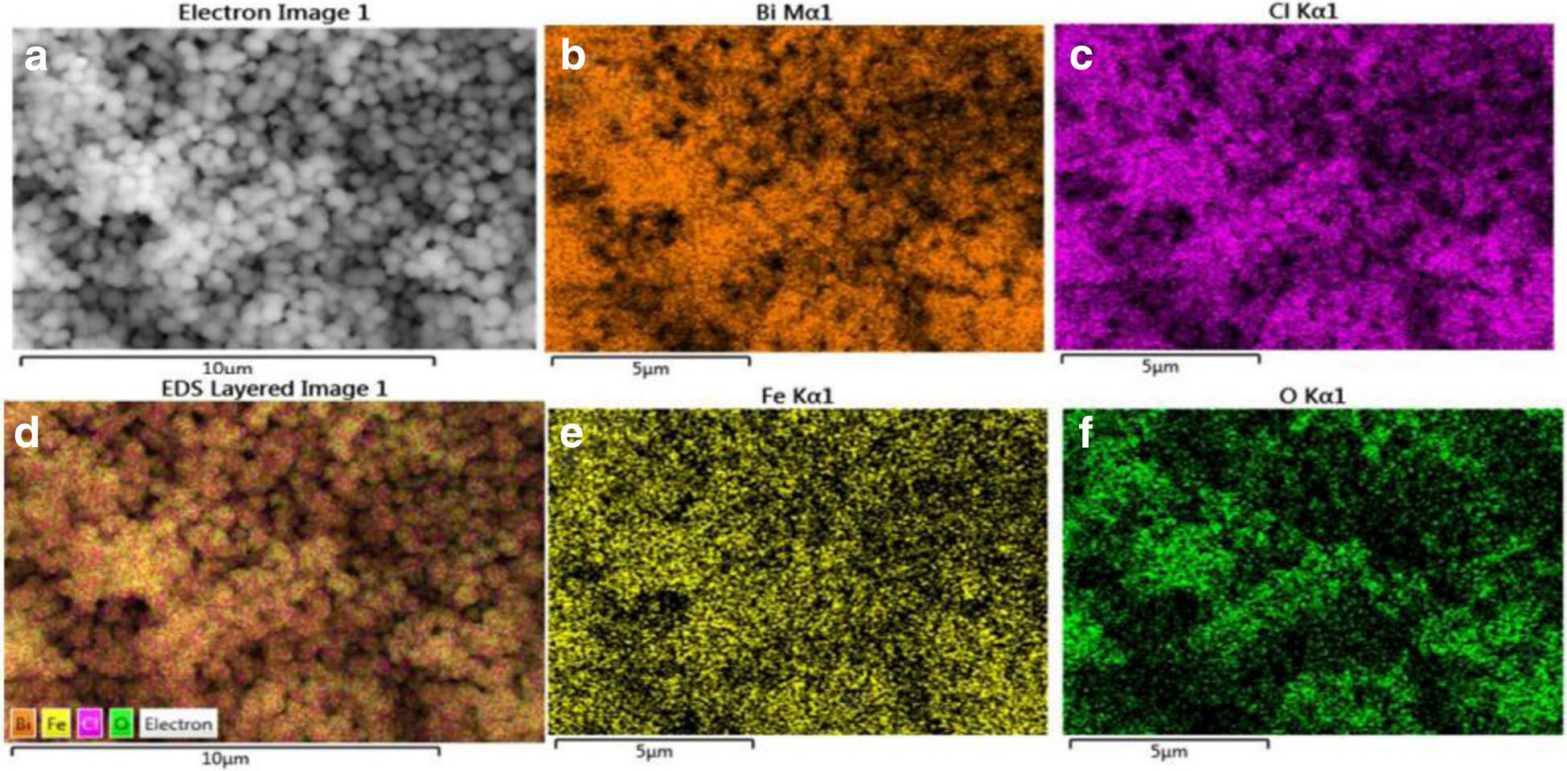
Typical SEM images (**a**) and the elemental mapping of Bi (**b**), Cl (**c**), all elements (**d**),  Fe (**e**), and O (**f**) of Fe/BiOCl (0.25)

Based on the results that no apparent peak shifts and no new diffraction peaks related to Fe species are observed in XRD patterns as well as the same *d* spacings of BiOCl and Fe/BiOCl, it could be concluded that Fe^3+^ ions are not detected in our Fe/BiOCl samples. To further prove the presence of Fe^3+^ ions, the elemental mapping of Fe/BiOCl (0.25) was conducted by SEM with an energy dispersive X-ray (EDX), which result is shown in Fig. 4. As can be seen in Fig. 4, the Fe element is homogeneously distributed on the surface of Fe/BiOCl micro-flowers, which forcefully evidences the existence of Fe^3+^ ions.

**Fig. 5:**
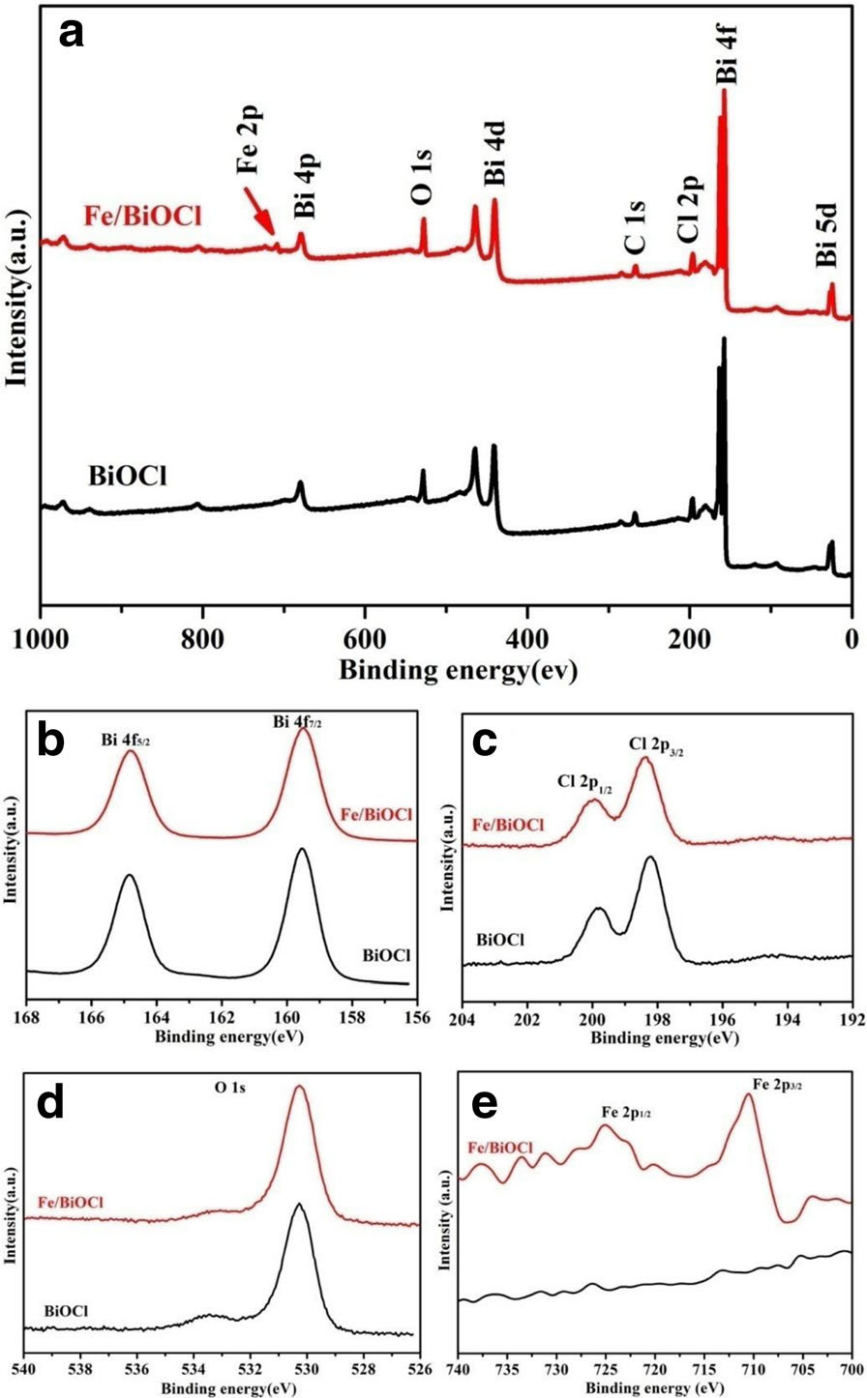
The XPS spectra of BiOCl and Fe/BiOCl (0.25). **a** Survey, **b** Bi 4f, **c** Cl 2p, **d** O 1s, and **e** Fe 2p

In order to determine the chemical compositions and specify the chemical states of surface elements in our samples, XPS measurement was further carried out (Fig. 5). The survey spectrum of sample Fe/BiOCl (0.25) mostly resembles that of bare BiOCl, which shows the co-presence of Bi, O, Cl, and C elements, except for a weak peak assigned to Fe signal in the range of 700~750 eV. The C peak is from the adventitious carbon on the surface of the sample. The high-resolution spectra of Bi 4f (Fig. 5b) display two intense peaks located at 164.8 and 159.5 eV, which are assigned to Bi 4f_5/2_ and Bi 4f_7/2_, respectively. As illustrated in the Cl 2p core level spectra (Fig. 5c), there are two clear peaks located at 198.2 and 199.8 eV, which correspond to Cl 2p_3/2_ and Cl 2p_1/2_, respectively. The binding energies of 530.3 and 533.4 eV in Fig. 5d are respectively assigned to the lattice oxygen in BiOCl or Fe/BiOCl and the defect-oxide and hydroxyl-like groups [41]. As depicted in Fig. 5e, two obvious peaks observed at 724.0 and 710.5 eV are assigned to Fe 2p_1/2_ and Fe 2p_3/2_, respectively. The energy span between the two levels is about 13 eV, which is a characteristic value for Fe^3+^ state [11, 42]. It should be noticed that there are no critical shifts of peak locations observed in Bi 4f, Cl 2p, and O 1s spectra after Fe^3+^ modification, indicating that the Fe^3+^ ions were just grafted on the surface of BiOCl or present as amorphous FeO(OH)-like clusters [43]. Particularly, the XPS technique could be also adopted to detect the chemical compositions of surface elements. Based on the XPS results, the Fe/Bi molar ratio of sample Fe/BiOCl (0.25) was estimated to be 0.27, which is very close to the original arranged value.

**Fig. 6:**
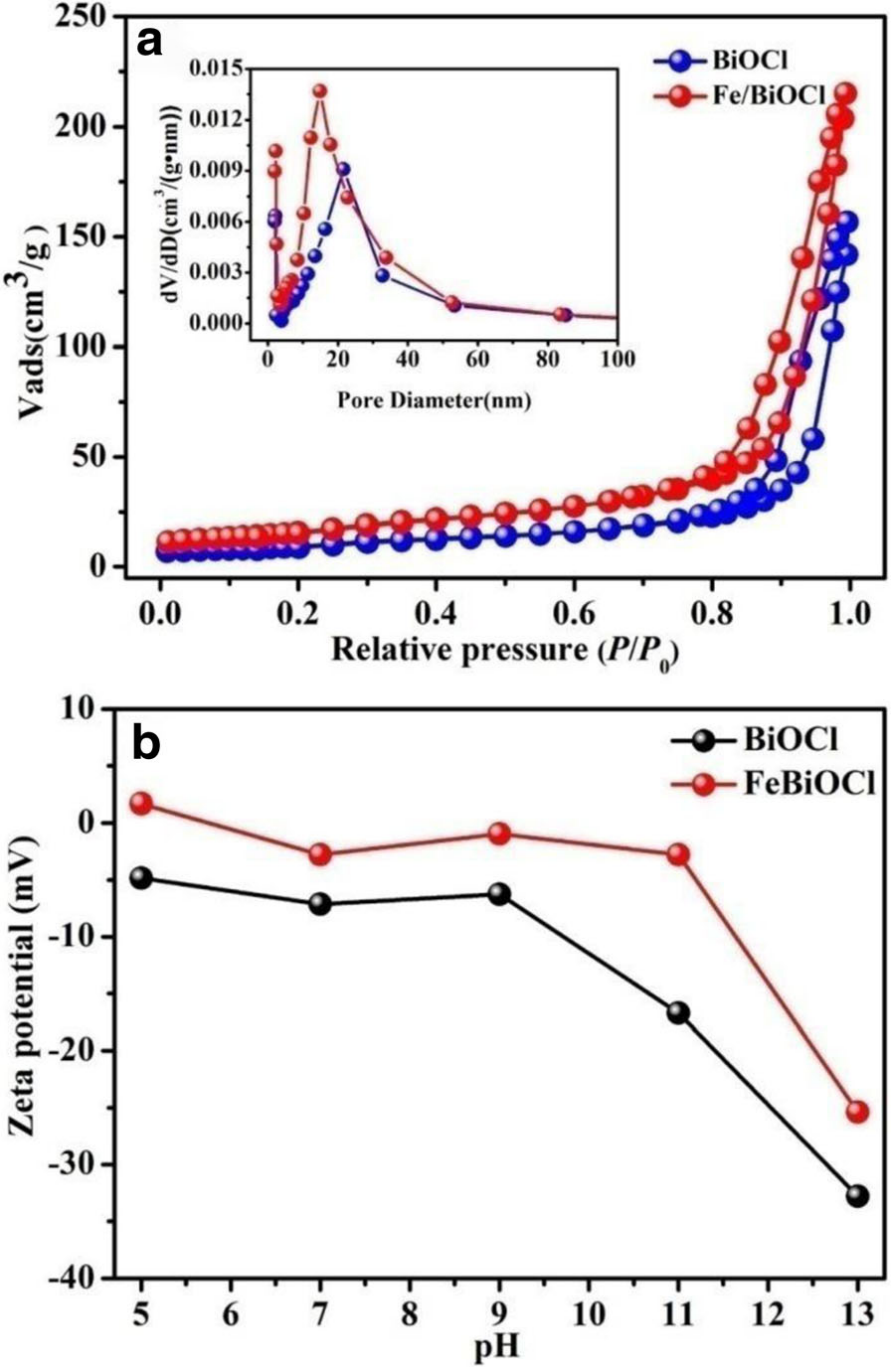
N_2_ adsorption-desorption isotherms as well as pore size distribution curves (insert) (**a**) and zeta potentials (**b**) of BiOCl and Fe/BiOCl (0.25)

Surface area is a crucial factor for adsorbents to remove toxic dyes, organic contaminants, and metal ions [28, 33, 44]. Higher specific surface area (*S*_BET_) and pore volume (*V*_T_) of an adsorbent could favor the sorption capacity [33]. Hence, the specific surface area as well as BJH pore size distribution were measured by N_2_ adsorption-desorption experiments and the results are shown in Fig. 6a and Table 1. Both of the N_2_ adsorption-desorption isotherms for samples BiOCl and Fe/BiOCl (0.25) are classified as type IV with H3 hysteresis loops, which demonstrates the existence of porous structure formed between each inter-crossed nanoplates or nanosheets [45, 46]. The BJH pore size distribution curves (inset in Fig. 6a) further confirm the presence of the porous structures in our samples. As listed in Table 1, the *S*_BET_ and *V*_T_ values of Fe/BiOCl (0.25) are higher than those of BiOCl, which could be ascribed to the smaller particle size and open microstructures after Fe^3+^ modification.

**Table 1:** Pore parameters of BiOCl and Fe/BiOCl (0.25)

Sample	*S*_BET_ (m^2^/g)	*V*_T_ (cm^3^/g)	Pore size (nm)
BiOCl	35.05	0.17	21.56
Fe/BiOCl	58.96	0.25	18.97

Zeta potential is widely used for quantification of the surface charge magnitude of the particles dispersed in solution [18], which is another key factor for an adsorbent. Fig. 6b shows the zeta potential of BiOCl and Fe/BiOCl (0.25) measured at various pH values. As shown in Fig. 6b, the surface of BiOCl is negatively charged among the pH value of 5~13. After Fe^3+^ modification, the charge is positively improved but still below 0 mV among the measured pH values. The positive improvement for surface charge is possibly ascribed to the charge neutralization via specific adsorption of Fe^3+^ ions onto the surface of BiOCl or the formation of hydroxyl groups (Fe-OH) which are protonated to form Fe-OH_2_^+^ [35].

### Adsorption Capacity of BiOCl and Fe/BiOCl

In the following adsorption experiment, Fe/BiOCl (0.25) was selected as the representative sample to check the adsorption performance of Fe/BiOCl (*x*).

**Fig. 7:**
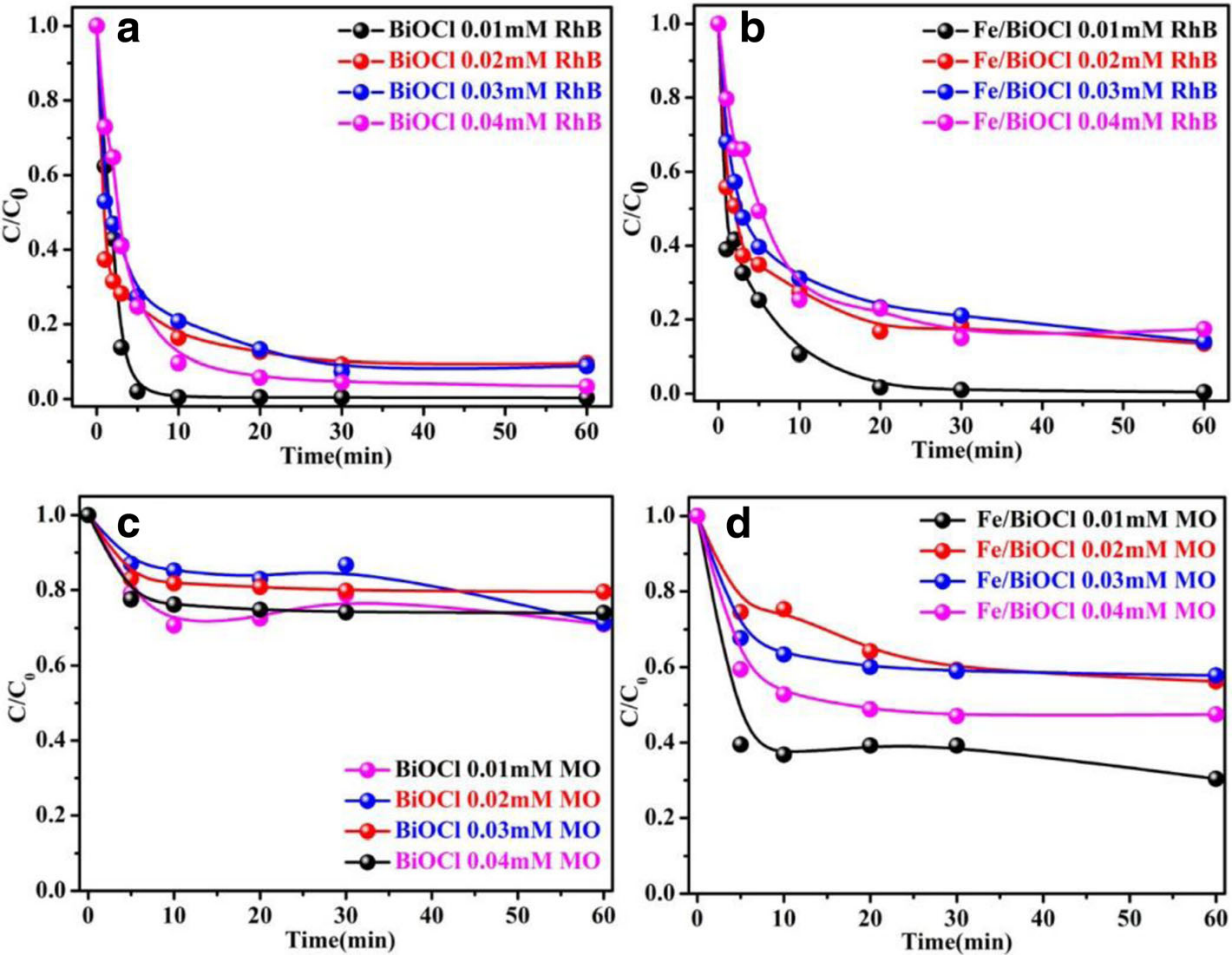
Effect of initial concentration on adsorption capacities of BiOCl and Fe/BiOCl toward RhB (**a**, **b**) and MO (**c**, **d**) (pH = 7, temperature = 25 °C)

Cationic dye RhB and anionic dye MO are chosen as the typical organic dyes to test the adsorption capacities of BiOCl and Fe/BiOCl. Figure 7 shows the adsorption capacities of BiOCl and Fe/BiOCl as a function of initial concentration of RhB and MO with an increase of time in the range of 0.01~0.04 mmol/L. As shown in Fig. 7, the adsorption capacities of BiOCl and Fe/BiOCl are time-dependent within 20 min and decrease with an increase of initial concentration of RhB and MO. The higher adsorption efficiency at lower concentration is possibly related to the fact that a maximum number of dye molecules are prone to adsorb on as-prepared adsorbents [47]. Figure 7a, b reveals that both BiOCl and Fe/BiOCl display excellent adsorption efficiency toward cationic dye RhB and could reach a maximum within 5 and 20 min for BiOCl and Fe/BiOCl, respectively. However, BiOCl shows poor adsorption performance toward anionic dye MO due to the increasing electrostatic repulsion, which is only about 30% adsorption efficiency within 20 min. After Fe^3+^ modification, the adsorption capacity is enhanced, which reaches about 60% within 20 min. The enhanced adsorption capacity toward anionic MO may be related to the more open porous structure and higher specific surface area of Fe/BiOCl. Thus, it could be deduced that BiOCl and Fe/BiOCl are remarkable for removing the RhB but not suitable adsorbents for MO.

**Fig. 8:**
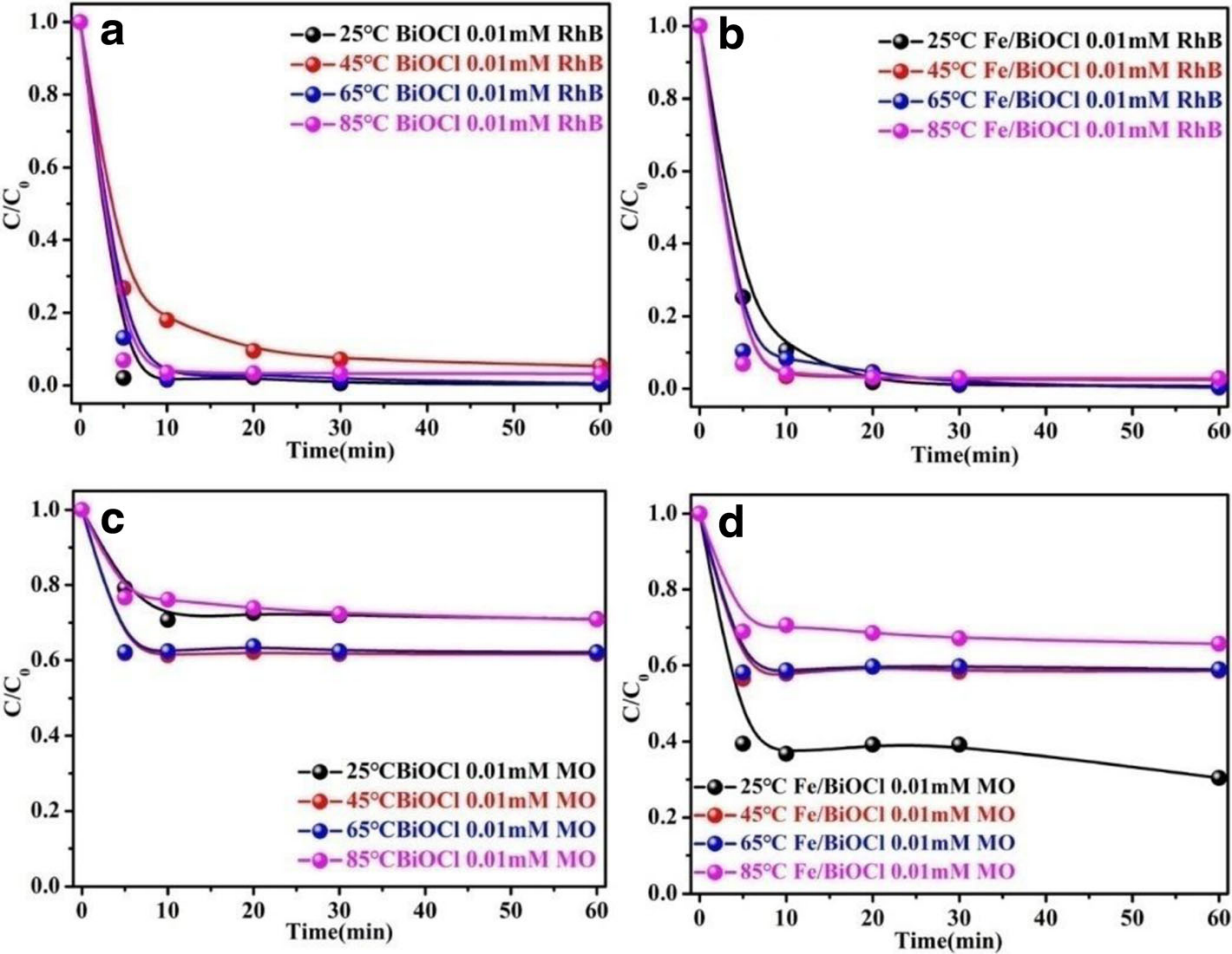
Effect of temperature on adsorption capacities of BiOCl and Fe/BiOCl toward RhB (**a**, **b**) and MO (**c**, **d**) (pH = 7, initial concentration = 0.01 mmol/L)

The effect of temperature (25~85 °C) on the adsorption capacities of BiOCl and Fe/BiOCl toward RhB and MO was also investigated. The results shown in Fig. 8 demonstrate that there is not a close connection between the adsorption capacities of BiOCl as well as Fe/BiOCl and temperature for removing RhB; however, the adsorption capacities of BiOCl and Fe/BiOCl are highly dependent on the temperature toward MO, and the low temperature is favorable for this adsorption process. Moreover, the adsorption capacities of BiOCl and Fe/BiOCl toward RhB are still higher than the values toward MO, which result is consistent with Fig. 7. Based on the results from Fig. 7, the electrostatic attraction between dye molecules and adsorbents is mainly responsible for the large adsorption capacity of BiOCl. After Fe^3+^ modification, the surface of BiOCl is more positively charged (Fig. 6b), which result is analogous to that of Fe^3+^-grafting clinoptilolite [35]. This phenomenon is disadvantageous for removing the cationic dyes from solutions. Nevertheless, the adsorption capacities of Fe/BiOCl toward RhB nearly remains consistent with the values of BiOCl in the temperature range of 25~85 °C. As known, higher specific surface area could provide more active sites for dye molecules adsorption [33, 35, 48]. The specific surface area of Fe/BiOCl (58.96 m^2^/g) is higher than that of BiOCl (35.05 m^2^/g); thus, the specific surface area also plays an important role in the adsorption process of dye molecules on Fe/BiOCl.

**Fig. 9:**
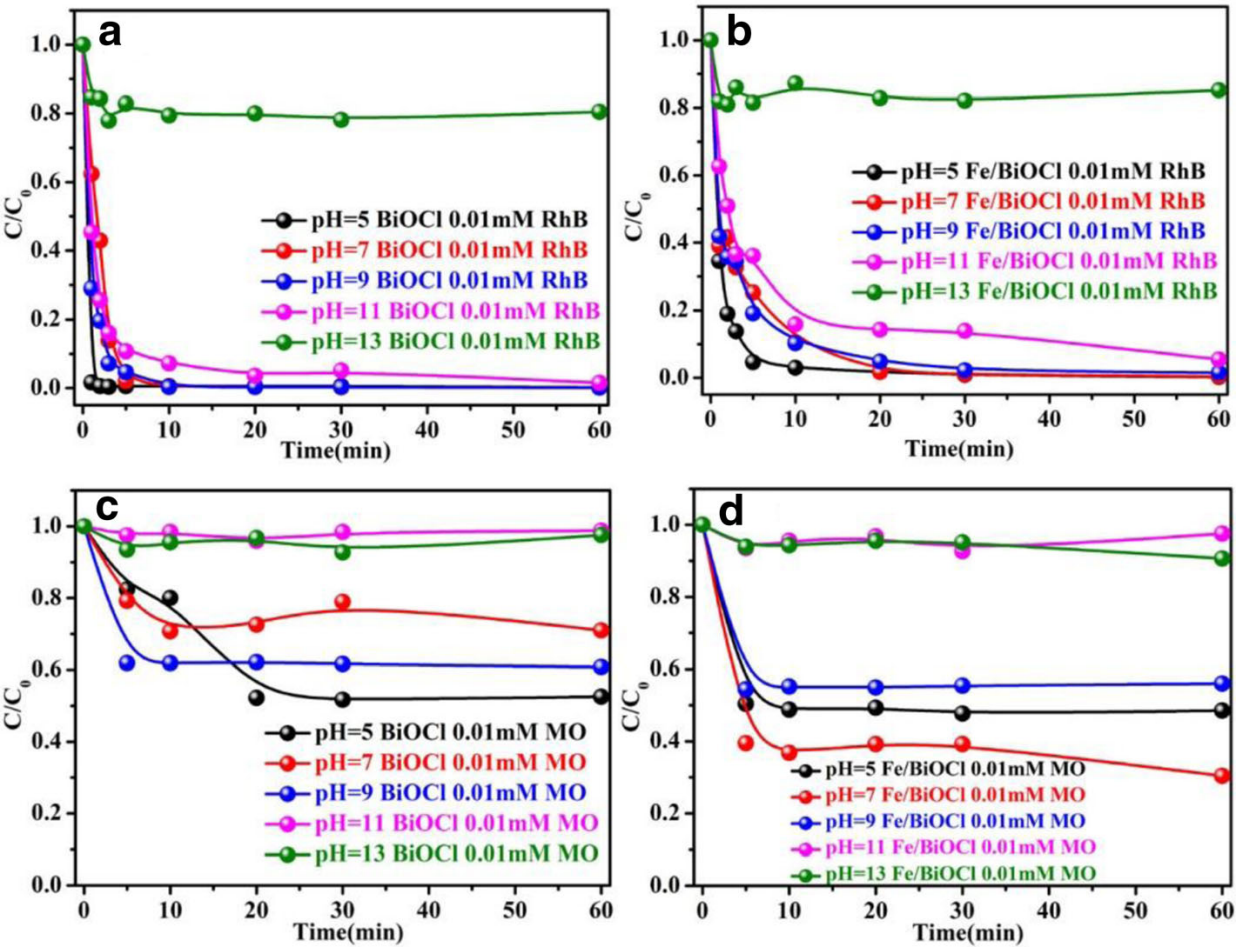
Effect of pH value on adsorption capacities of BiOCl and Fe/BiOCl toward RhB (**a**, **b**) and MO (**c**, **d**) (temperature = 25 °C, initial concentration = 0.01 mmol/L)

The pH value of solution acts a vital role in controlling the interactions between the adsorbent and the dye molecules, because both the surface charge of adsorbent and the ionization degree of dye molecules are highly affected by the solution pH [7]. The effect of pH value in the range of 5~13 adjusted by 0.1 M HCl or 0.1 M NaOH on adsorption capacities was also studied and the results are shown in Fig. 9. The adsorption capacities of BiOCl and Fe/BiOCl toward RhB and MO are strongly pH dependent, which display poor adsorption performance in the alkaline solution. Figure 9a, b shows that the initial rate of dye molecule uptake increased sharply to 99.7% within 3 min on BiOCl and 93.0% within 10 min on Fe/BiOCl and then leveled off with prolonging the time at pH value of 5. As known, there would be an adsorption competition between H^+^ and cationic RhB molecules in the acidic solution [47, 49]. However, the adsorption capacities of BiOCl and Fe/BiOCl toward RhB display no decrease compared with the values at pH = 7, which indicates that there is no adsorption competition between H^+^ and cationic RhB molecules. It is generally accepted that the alkaline solution is beneficial for the enhancement of adsorption capacities of BiOCl and Fe/BiOCl toward cationic dyes, because both BiOCl and Fe/BiOCl are negatively charged (Fig. 6b) and there is no adsorption competition between OH^−^ and dye molecules. Unfortunately, the adsorption capacities of BiOCl and Fe/BiOCl toward RhB sharply decreased at pH = 13, which is possibly ascribed to the structural destruction of BiOCl because BiOCl is unstable in the strong alkaline solution [50]. The adsorption behavior of MO on BiOCl and Fe/BiOCl resembles that of RhB, i.e., the adsorption capacities in acidic solution are higher than the values in the alkaline solution. The difference is that the adsorption capacities of BiOCl and Fe/BiOCl toward MO sharply decrease at pH = 11, which phenomenon may be related to the weak adsorption competition between OH^−^ and anionic MO molecules [35, 51].

Based on the above adsorption experiments, the maximum adsorption capacities of BiOCl and Fe/BiOCl toward RhB is optimized in the condition of initial concentration = 0.01 mmol/L, pH value = 5.0, and temperature = 25 °C.

### Adsorption Mechanism

Based on the zeta potential and above adsorption results, we can infer that the strong electrostatic attraction plays a major role in the adsorption process. To confirm this deduction, other two organic dyes including cationic methylene blue (MB) and anionic acid orange (AO) are chosen to further investigate the adsorption performances of BiOCl and Fe/BiOCl. Additional file 1: Figure S1 shows the adsorption efficiencies of MB and AO on BiOCl and Fe/BiOCl. As displayed in Additional file 1: Figure S1, both BiOCl and Fe/BiOCl exhibit excellent adsorption efficiencies toward cationic MB but disappointing performance toward anionic AO, which result turns out the assumption that the strong electrostatic attraction is primarily responsible for the adsorption performances of BiOCl and Fe/BiOCl.

Other than the strong electrostatic attraction, the high specific surface area and open porous structure also contribute to the adsorption performances of as-prepared adsorbents. Generally speaking, the Fe^3+^ grafting makes the surface of BiOCl more positively charged (Fig. 6b) than parent BiOCl, which would induce the decreased adsorption capacity of Fe/BiOCl toward cationic dye molecules. However, the adsorption capacity of Fe/BiOCl nearly maintains the same value as that of BiOCl. Furthermore, Fe/BiOCl shows higher adsorption capacity toward anionic dye molecules than bare BiOCl, although both of them are negatively charged. It should be noticed that Fe/BiOCl has a more open porous structure and higher specific surface area (TEM and BET results) than parent BiOCl, both of which are favorable for enhancing the adsorption capacity. Thus, it could be deduced that three parameters, including the electrostatic attraction, higher specific surface area, and more open porous structure are responsible for the adsorption capacity of Fe/BiOCl.

In a conclusion, the adsorption mechanism of BiOCl and Fe/BiOCl toward organic dyes could be summarized as follows: (1) For the adsorbent BiOCl, strong electrostatic attraction is the major reason for the adsorption capacity toward cationic dye molecules, but porous structure and high specific surface area are mainly responsible for the adsorption capacity toward anionic dye molecules; (2) For the adsorbent Fe/BiOCl, three aspects containing electrostatic attraction, more open porous structure, and higher specific surface area are responsible for the adsorption capacity toward cationic dye molecules, but the latter two aspects are the main reasons for the adsorption capacity toward anionic dye molecules.

### Adsorption of Mixed Dyes on BiOCl and Fe/BiOCl

**Fig. 10:**
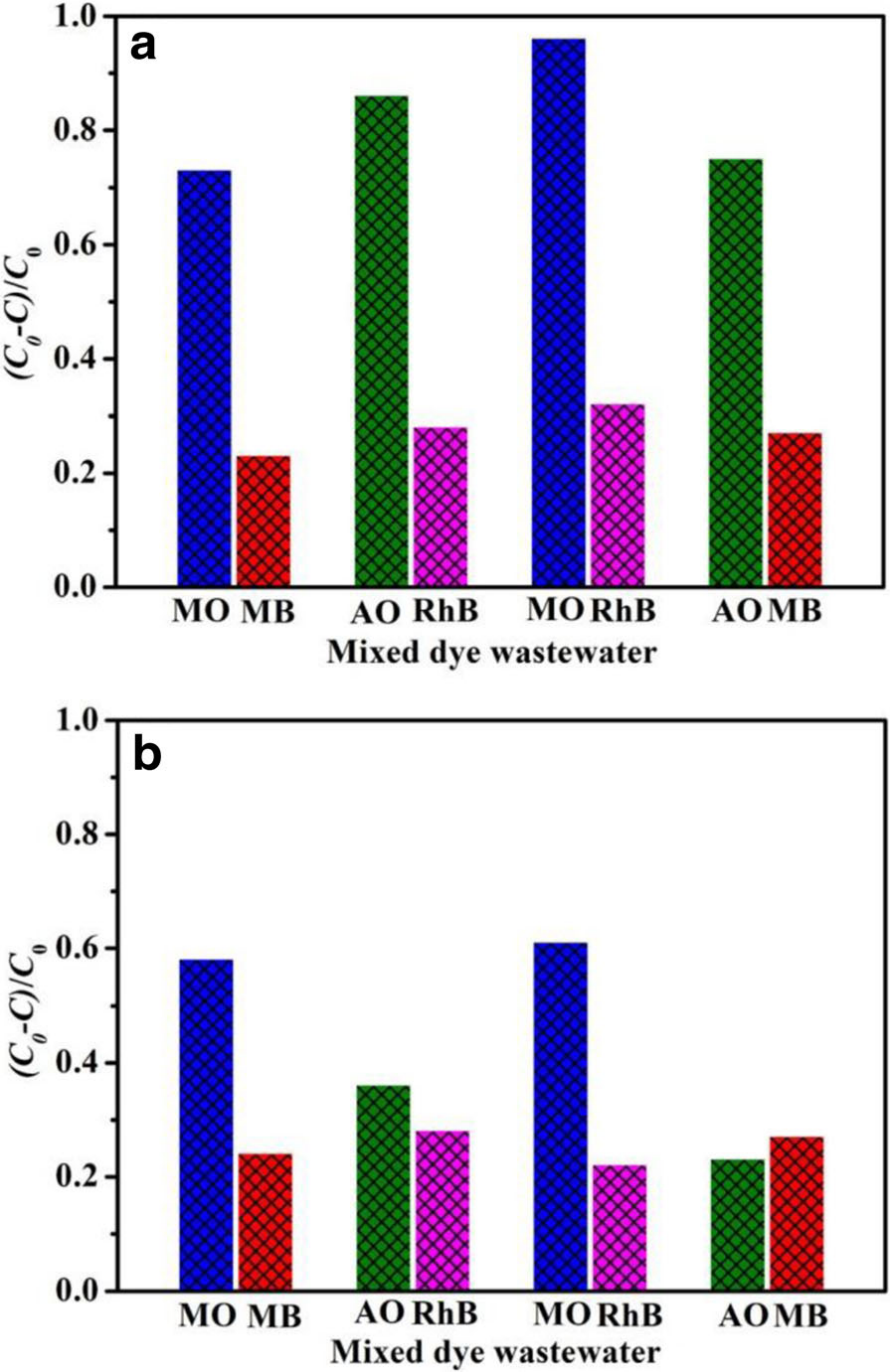
Adsorption capacities of MO, MB, RhB, and AO in mixed dye solutions on BiOCl (**a**) and Fe/BiOCl (**b**). Adsorption condition 50 mg adsorbent, 25 mL of single dye in mixed dye solution, room temperature. All the concentration of dye solutions is 0.01 mmol/L

The actual industrial dye wastewater is usually composed of more than one kind of dyes. Therefore, a series of mixed dye solutions is prepared to examine the adsorption performance of as-prepared adsorbents. Figure 10 displays the selective adsorption performances of BiOCl and Fe/BiOCl toward mixed dye solutions, and the selective adsorption capacities of dye molecules as a function of time for BiOCl and Fe/BiOCl (0.25) are shown in Additional file 1: Figures S2 and S3, respectively. The results reveal that BiOCl shows more excellent selective adsorption performance than Fe/BiOCl toward cationic dye molecules in the mixed dye solutions. However, the adsorption capacities of various dye molecules are generally lower than those of the corresponding single dye systems, which is possibly resulted by the competitive adsorption of dye molecules on the surface of adsorbents [7].

### Adsorption Isotherms

Adsorption isotherm is often adopted to determine the equilibrium relationship between the adsorbent and the dye molecules as well as the equilibrium concentration of the dye molecules [52]. Langmuir isotherm and Freundlich isotherm are the most frequently used isotherms; the former model is based on the assumption that the maximum adsorption capacity keeps a correspondence with a saturated monolayer of solute molecules on the adsorbent surface, and the latter model describes a kind of multilayer adsorption with the solutes from a liquid to a solid surface and provides a relationship between the adsorbed dye amounts and the dye concentration at equilibrium [48, 49, 52]. The linear form of the Langmuir equation can be described as follows:

3 $$ \frac{C_e}{q_e}=\frac{1}{Q_0b}+\frac{C_e}{Q_0} $$

where *C*_*e*_ (mg/L) is the equilibrium concentration of the dye molecules, *q*_*e*_ (mg/g) is the amount of adsorbed dyes per unit mass of adsorbent at equilibrium, and *Q*_0_ and *b* are the Langmuir constants which are related to adsorption capacity and rate of the adsorption, respectively.

The Freundlich isotherm, an empirical equation, can be described as follows:

4 $$ \ln {q}_e=\frac{1}{n_F}\ln {C}_e+\ln {K}_f $$

where *q*_*e*_ (mg/g) is the amount of adsorbed dyes per unit mass of adsorbent at equilibrium, *C*_*e*_ (mg/L) is the equilibrium concentration of dye molecules, and *K*_*f*_ (L/mg) and *n*_*F*_ are Freundlich constants which are associated with the adsorption capacity at unit concentration and adsorption intensity of the adsorbent, respectively.

**Fig. 11:**
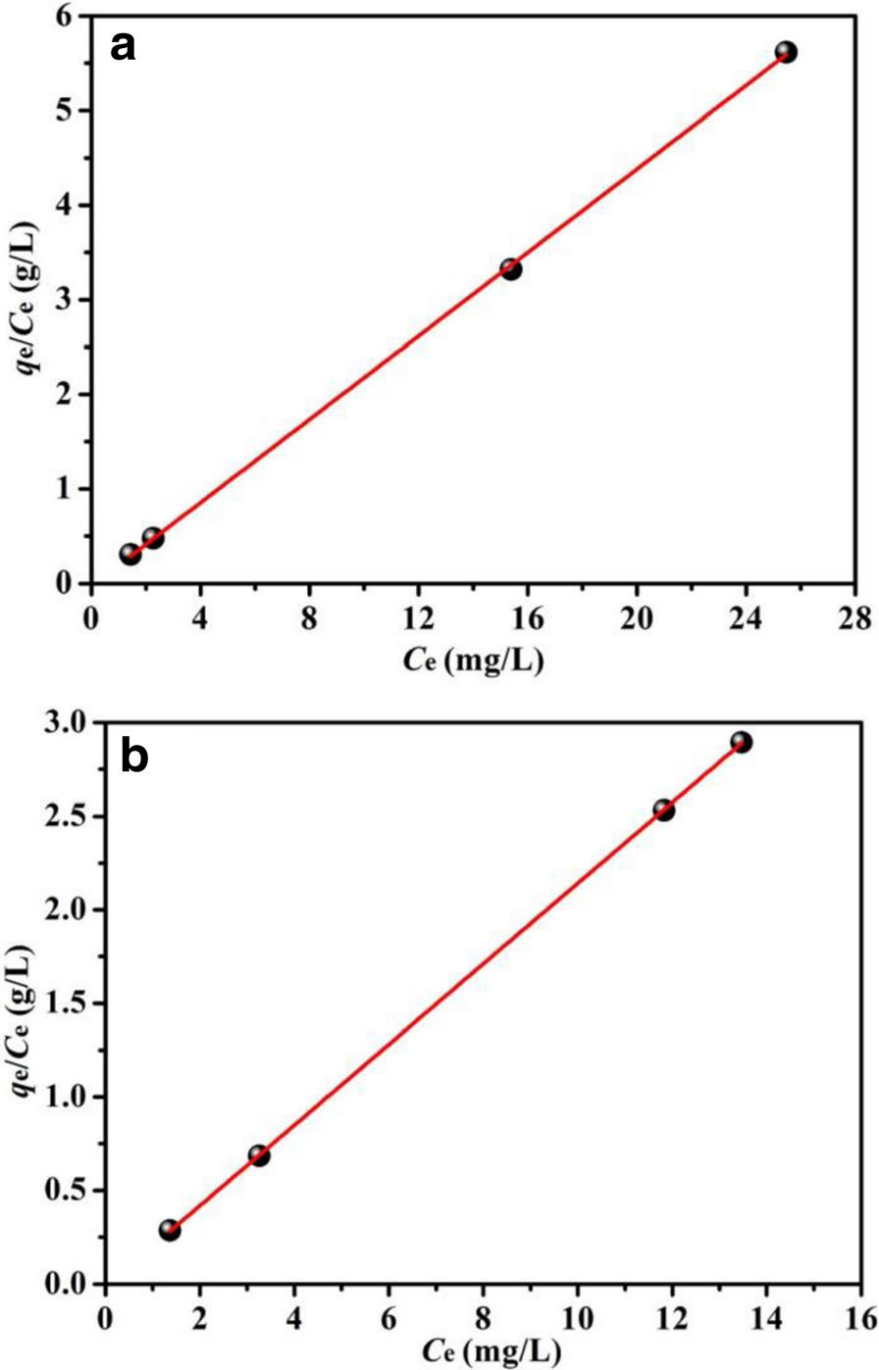
Langmuir isotherm for adsorption RhB on BiOCl (**a**) and Fe/BiOCl (**b**)

The plots of the experimental data on the basis of Langmuir and Freundlich models are shown in Fig. 11 and Additional file 1: Figure S4, respectively. It is obviously observed in Fig. 11 and Additional file 1: Figure S4 that the Langmuir isotherm model displays a better fit to the experimental data for both BiOCl and Fe/BiOCl than the Freundlich isotherm model, which indicates the monolayer coverage of the surface of BiOCl and Fe/BiOCl by RhB molecules.

### Adsorption Kinetics

To further investigate the adsorption rate and the possible mechanism, kinetics of RhB adsorption on BiOCl and Fe/BiOCl at different temperatures were studied using the pseudo-first order and the pseudo-second order [53, 54], respectively.

The pseudo-first order can be described as Eq. (5):

5 $$ \ln \left({q}_e-{q}_t\right)=\ln {q}_e-{k}_1t $$

The pseudo-second-order can be described as Eq. (6):

6 $$ \frac{t}{q_t}=\frac{1}{k_2{q}_e^2}+\frac{t}{q_e} $$

where *q*_*t*_ (mg/g) and *q*_*e*_ (mg/g) are the amount of dye molecules adsorbed at *t* time and at equilibrium, respectively. *k*_*1*_ (min^− 1^) and *k*_2_ (g/(mg min)) represent the rate constant of the pseudo-first-order model and the pseudo-second-order model, respectively.

**Fig. 12:**
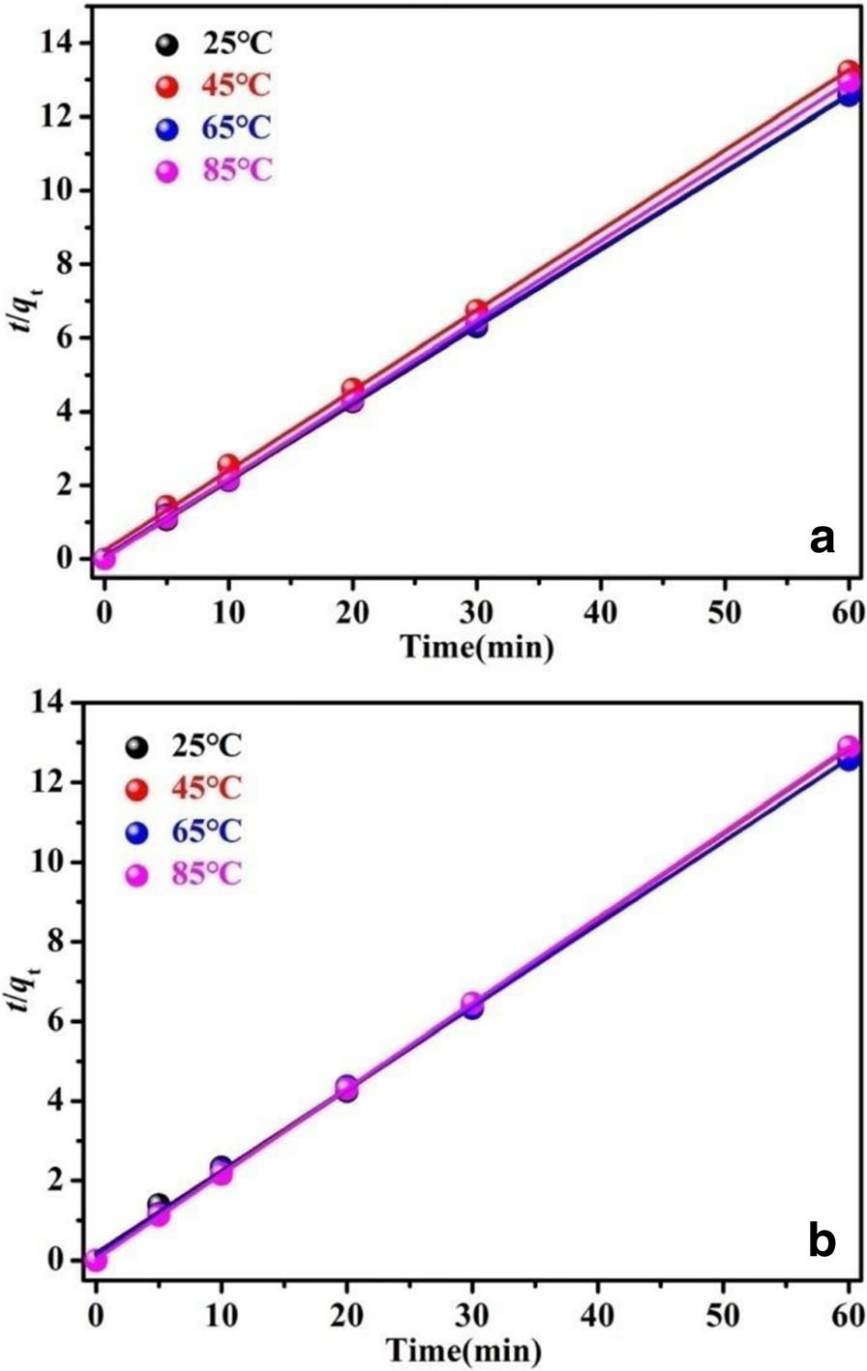
Pseudo-second-order kinetics for adsorption RhB on BiOCl (**a**) and Fe/BiOCl (**b**)

The plots of the experimental data simulated on the basis of the pseudo-first-order and the pseudo-second-order are shown in Additional file 1: Figure S5 and Fig. 12, respectively. As shown in Additional file 1: Figure S5 and Fig. 12, the experimental data shows a better fit to the pseudo-second-order model than the pseudo-first-order model. The values of kinetic parameters *q*_*e*_ and *k*_*2*_ and the corresponding correlation coefficients (*R*^2^) are listed in Additional file 1: Table S1. All the *q*_*e*_ values are very close to the theoretical value for complete adsorption capacity for RhB (4.79 mg/g), which indicates the forceful adsorption efficiency of BiOCl and Fe/BiOCl. The low *q*_*e*_ value is possibly resulted by the low concentration of as-prepared dye solutions.

### Adsorption Cycles and Adsorbent Regeneration

**Fig. 13:**
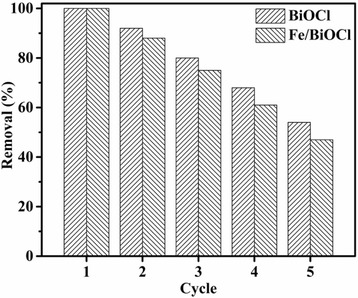
The adsorption cycle tests of BiOCl and Fe/BiOCl

For potential applications in pollutant treatment, the recycled utilization of an adsorbent plays a significant role. Thus, the adsorption cycle tests of BiOCl and Fe/BiOCl toward RhB were conducted and the results are shown in Fig. 13. As displayed in Fig. 13, the adsorption efficiency of BiOCl maintained more than 80% after three adsorption cycles. The adsorbent Fe/BiOCl also presented excellent adsorption efficiency, i.e., about 50% after five adsorption cycles, although which was slightly lower than that of BiOCl.

It is generally accepted that BiOCl is recognized as an excellent photocatalyst toward organic dye photodegradation [22–24]. Thus, this photocatalytic performance could be applied to regenerate the adsorbents. Using RhB as reaction model, the regeneration of BiOCl and Fe/BiOCl was investigated and the detailed description was displayed in Additional file 1. Additional file 1: Figure S6 (a) showed the FT-IR spectra of RhB, BiOCl, and Fe/BiOCl and the corresponding counterparts after adsorption and photocatalytic process. A series of bands at 1000–1800 cm^− 1^ are attributed to RhB dye molecules [55], and the peak at 522 cm^− 1^ is attributed to the Bi–O stretching vibration [56]. After adsorption of RhB dye molecules onto BiOCl and Fe/BiOCl, many peaks belonging to RhB were observed and Bi–O stretching vibration did not changed, which confirmed the electrostatic interaction between adsorbents and RhB molecules as well as the high stability of adsorbents. In addition, the photocatalytic activities of BiOCl and Fe/BiOCl after adsorption were measured under visible light illuminations. After 60 min irradiation, the residual samples were collected and washed with water. It is noticeable that the characteristic peaks of functional groups for RhB molecules became very weak in samples BiOCl and Fe/BiOCl, forcefully demonstrating the regeneration and superior photocatalytic activities of absorbents. Additional file 1: Figure S6 (b) shows the intuitive photographs of as-prepared BiOCl and Fe/BiOCl and the corresponding samples after adsorption and photodegradation. The pristine BiOCl and Fe/BiOCl displayed white and light brown colors, which turned to nearly RhB color after adsorption and then approximately faded into the original color of samples after photodegradation. The color variation of the adsorbents verifies the adsorption and photodegradation of RhB over BiOCl and Fe/BiOCl, further confirming that BiOCl and Fe/BiOCl are excellent adsorbents and could be easily regenerated by a photocatalytic route.

## Conclusions

In summary, two adsorbents including BiOCl and Fe/BiOCl were prepared for the removal of cationic and anionic dyes with low concentration from the solutions. After grafting Fe^3+^ on the surface of BiOCl, the adsorbent showed more open porous structure and higher specific surface area. Both BiOCl and Fe/BiOCl are more favorable for removing the cationic dye molecules from the solution, whereas Fe/BiOCl displays higher adsorption capacity toward anionic dye molecules than BiOCl. Furthermore, BiOCl exhibited higher selective adsorption efficiency toward cationic dye molecules than Fe/BiOCl in mixed dye solutions. The prominent adsorption efficiency is probably to provide a potential application for as-prepared adsorbents in actual industrial wastewater.

## Additional file

Additional file 1: **Figure S1.** Adsorption capacities of BiOCl and Fe/BiOCl toward MB (a) and AO (b). **Figure S2.** Adsorption capacities of MO, MB, RhB, and AO as a function of time in mixed dye solutions on BiOCl. **Figure S3.** Adsorption capacities of MO, MB, RhB, and AO as a function of time in mixed dye solutions on Fe/BiOCl. **Figure S4.** Freundlich isotherm for adsorption RhB on BiOCl (a) and Fe/BiOCl (b). **Figure S5.** Pseudo-second-order kinetics for adsorption RhB on BiOCl (a) and Fe/BiOCl (b). **Table S1.** Parameters based on the pseudo-second-order kinetics for adsorption RhB on BiOCl and Fe/BiOCl. **Figure S6.** FT-IR spectra (a) and photographs of various samples (1-RhB, 2-BiOCl, 3-Fe/BiOCl, 4-BiOCl after adsorption, 5-Fe/BiOCl after adsorption, 6-BiOCl after adsorption and photodegradation, 7-Fe/BiOCl after adsorption and photodegradation). (DOCX 669 kb)

## Abbreviations

AO: Acid orange
BET: Brunauer-Emmett-Teller
BiOCl: Bismuth oxychloride
BJH: Barrett-Joyner-Halenda
Fe/BiOCl: Fe^3+^-grafted BiOCl
HRTEM: High-resolution TEM
MB: Methylene blue
MO: Methyl orange
RhB: Rhodamine B
*S*_BET_: Specific surface area
SEM: Scanning electron microscopy
TEM: Transmission electron microscopy
*V*_T_: Pore volume
XPS: X-Ray photoelectron spectroscopy
XRD: X-ray powder diffraction
