# Supplementary material for: Synthesis and Characterization of Modified BiOCl and Their Application in Adsorption of Low-Concentration Dyes from Aqueous Solution
Source: Nanoscale Res Lett. 2018 Mar 1;13:69. doi: 10.1186/s11671-018-2480-y (PMC5834949; doi:10.1186/s11671-018-2480-y)
Supplement: Supplementary file 1 — Figure S1. Adsorption capacities of BiOCl and Fe/BiOCl toward MB (a) and AO (b). Figure S2. Adsorption capacities of MO, MB, RhB, and AO as a function of time in mixed dye solutions on BiOCl. Figure S3. Adsorption capacities of MO, MB, RhB, and AO as a function of time in mixed dye solutions on Fe/BiOCl. Figure S4. Freundlich isotherm for adsorption RhB on BiOCl (a) and Fe/BiOCl (b). Figure S5. Pseudo-second-order kinetics for adsorption RhB on BiOCl (a) and Fe/BiOCl (b). Table S1. Parameters based on the pseudo-second-order kinetics for adsorption RhB on BiOCl and Fe/BiOCl. Figure S6. FT-IR spectra (a) and photographs of various samples (1-RhB, 2-BiOCl, 3-Fe/BiOCl, 4-BiOCl after adsorption, 5-Fe/BiOCl after adsorption, 6-BiOCl after adsorption and photodegradation, 7-Fe/BiOCl after adsorption and photodegradation). (DOCX 669 kb) [file 11671_2018_2480_MOESM1_ESM.docx]

**Supplementary Information**

**Synthesis and characterization of modified BiOCl and their application in adsorption of low-concentration dyes from aqueous solution**

Qihang Zhao^a^, Yongxing Xing^a^, Zhiliang Liu^a^, Jing Ouyang^b^, Chunfang Du^*a,b^

^a^*College of Chemistry and Chemical Engineering, Inner Mongolia University, Hohhot, Inner Mongolia, 010021, P. R. China.*

^b^*Hunan Key Laboratory of Mineral Materials and Application, Central South University, Changsha, 410083, P. R. China*

Qihang Zhao (qihang151@126.com) Yongxing Xing (yongxing766@163.com)

Zhiliang Liu (cezlliu@imu.edu.cn) Jing Ouyang (lhitxu@163.com)

*Corresponding author E-mail: cedchf@imu.edu.cn; Fax: +86-471-4994375; Tel: +86-471-4994375*


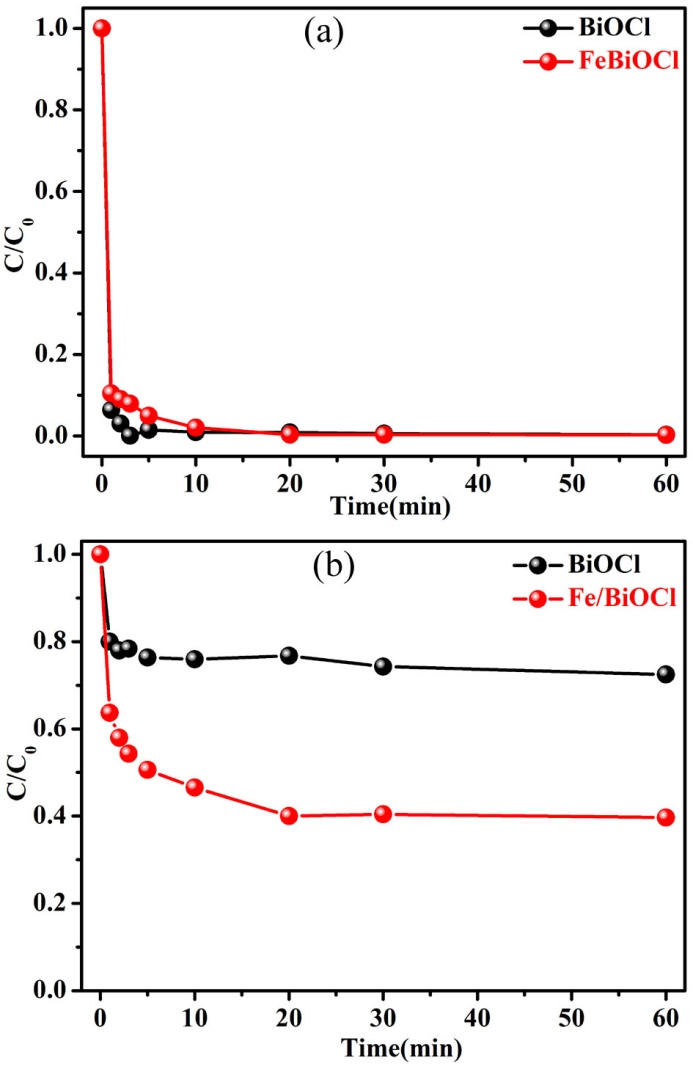


**Figure S1** Adsorption capacities of BiOCl and Fe/BiOCl toward MB (a) and AO (b)


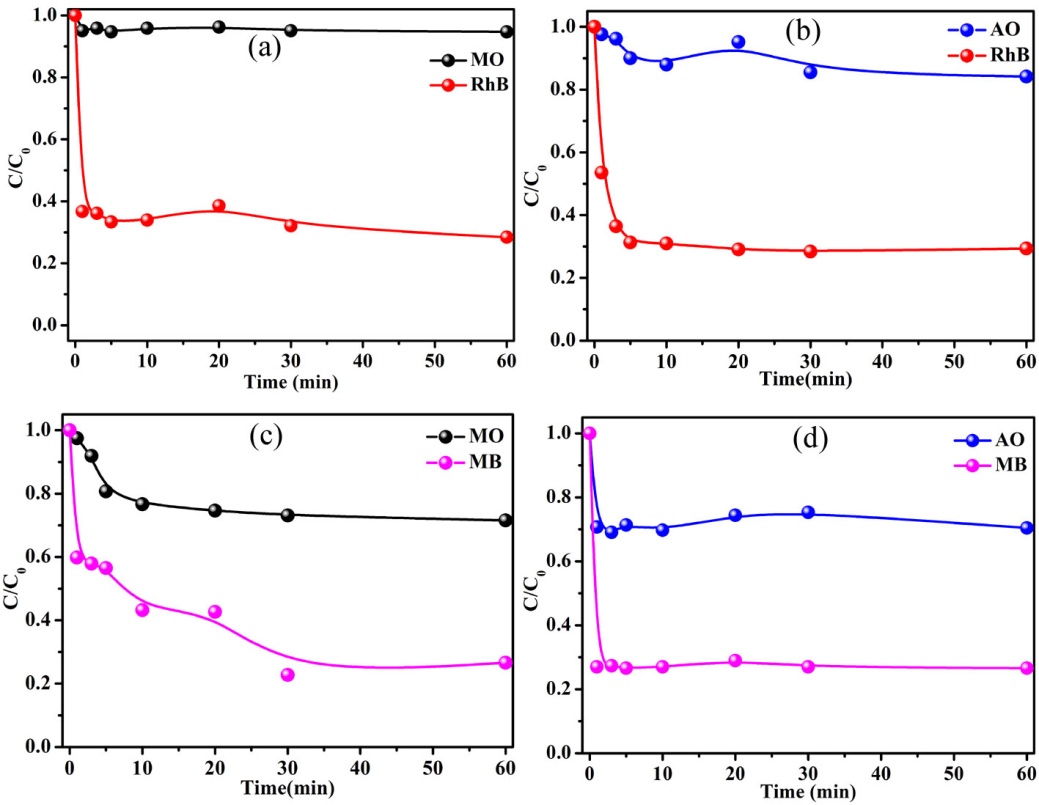


**Figure S2** Adsorption capacities of MO, MB, RhB and AO as a function of time in mixed dye solutions on BiOCl


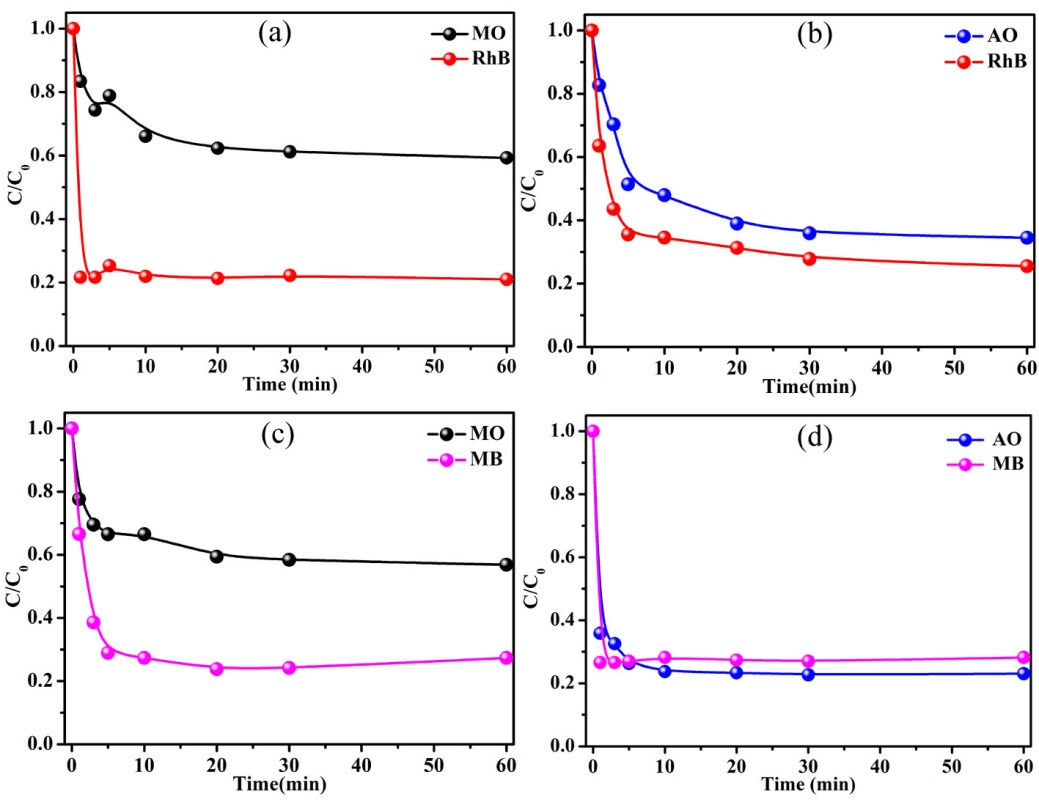


**Figure S3** Adsorption capacities of MO, MB, RhB and AO as a function of time in mixed dye solutions on Fe/BiOCl


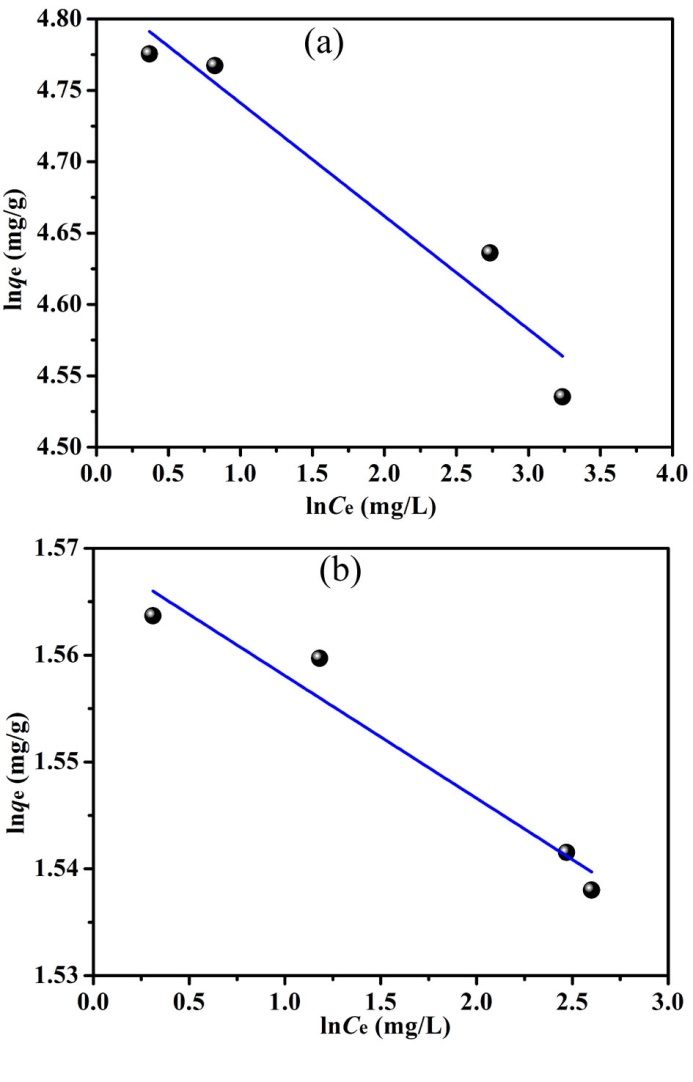


**Figure S4** Freundlich isotherm for adsorption RhB on BiOCl (a) and Fe/BiOCl (b)


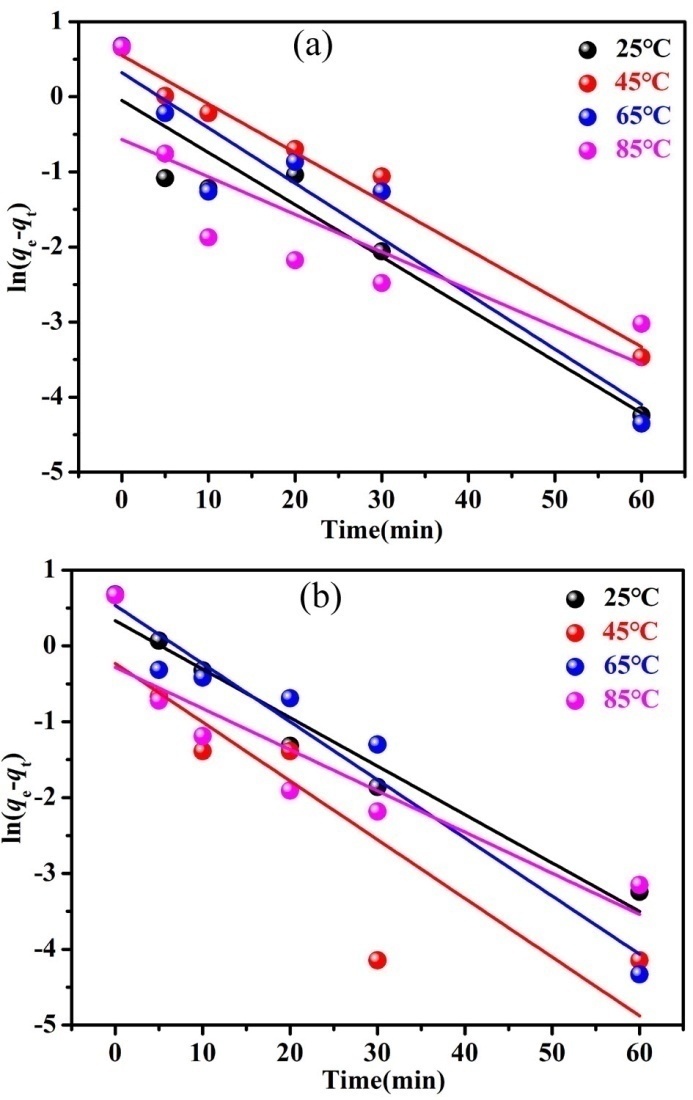


**Figure S5** Pseudo-second-order kinetics for adsorption RhB on BiOCl (a) and Fe/BiOCl (b)

**Table S1** Parameters based on the pseudo-second-order kinetics for adsorption RhB

on BiOCl and Fe/BiOCl

| *T* (ºC) | BiOCl | | | Fe/BiOCl | | |
| --- | --- | --- | --- | --- | --- | --- |
|  | *q*_e_ (mg/g) | *k*_2_ (g/(mg·min)) | *R*^2^ | *q*_e_ (mg/g) | *k*_2_ (g/(mg·min)) | *R*^2^ |
| 25 | 4.78 | 1.58 | 0.9999 | 4.78 | 1.62 | 0.9999 |
| 45 | 4.60 | 0.20 | 0.9991 | 4.68 | 1.69 | 0.9999 |
| 65 | 4.80 | 0.54 | 0.9998 | 4.83 | 0.35 | 0.9996 |
| 85 | 4.76 | 4.41 | 0.9999 | 4.67 | 1.91 | 0.9999 |

**
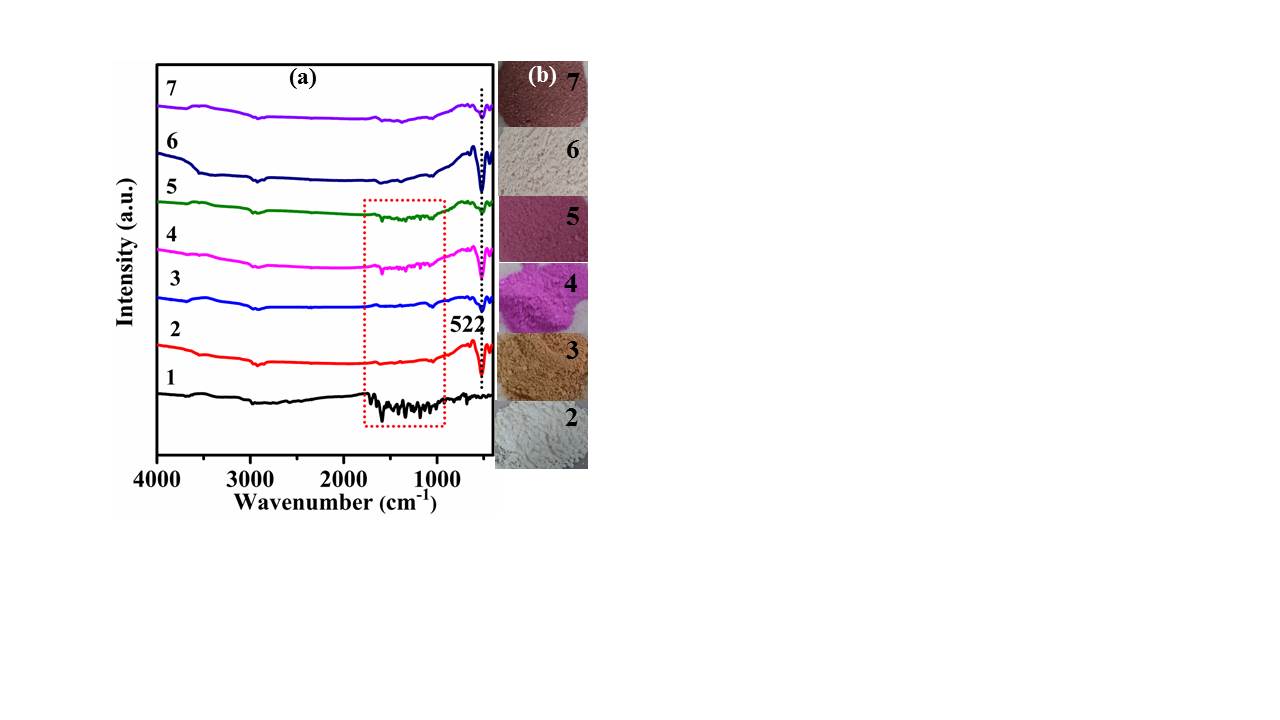
**

**Figure S6** FT-IR spectra (a) and photographs of various samples (1-RhB, 2-BiOCl, 3-Fe/BiOCl, 4-BiOCl after adsorption, 5-Fe/BiOCl after adsorption, 6-BiOCl after adsorption and photodegradation, 7- Fe/BiOCl after adsorption and photodegradation)

The adsorption and regeneration of BiOCl and Fe/BiOCl were conducted as follows. The adsorption process was similar to that conducted in the Experimental section. After adsorption for 1 hour, the generation of adsorbents was conducted by photocatalytic method. The photodegradation of RhB in aqueous solution was carried out in a 100 mL reactor under visible light irradiation. The suspension was illumined by Xe lamp with a UV cutoff filter (< 400 nm). After irradiation for another 1 hour, the suspension was centrifuged, washed with water several times and dried. Finally, various samples, including RhB, the pristine BiOCl and Fe/BiOCl as well as the corresponding counterparts after adsorption and photodegradation were analyzed by FT-IR and taken photographs.
